# Systolic pressure overload caused pulmonary oxidative stress, vessel remodeling and severe microvascular thrombosis in CD40 knockout mice through promoting platelet aggregation

**DOI:** 10.1016/j.redox.2026.104122

**Published:** 2026-03-21

**Authors:** Wenhui Yue, Yanyan Xu, Xinyu Weng, Dongzhi Wang, Linlin Shang, Haojie Jiang, Edward Kenneth Weir, Junling Liu, Yawei Xu, Wenliang Che, Yingjie Chen

**Affiliations:** a Department of Cardiology, Shanghai Tenth People's Hospital, Tongji University School of Medicine, Shanghai, 200072, China; b Ruijin Hospital, Department of Laboratory Medicine, Shanghai Jiao Tong University School of Medicine, Shanghai, 200025, China; c College of Health Sciences and Technology, Key Laboratory of Cell Differentiation and Apoptosis of Chinese Ministry of Education, Shanghai jiao Tong University School of Medicine, Shanghai, 200025, China; d Department of Cardiology, Shanghai Institute of Cardiovascular Diseases, Zhongshan Hospital, Fudan University, Shanghai, 200025, China; e Lillehei Heart Institute and Department of Medicine, University of Minnesota Medical School, Minneapolis, MN, 55455, USA; f Department of Physiology & Biophysics, University of Mississippi Medical Center, Jackson, MS, 39216, USA; g Department of Biochemistry and Molecular Cell Biology, Shanghai Jiao Tong University School of Medicine, Shanghai, 200025, China

**Keywords:** Pulmonary thrombosis, Heart failure, Inflammation, Platelet dysfunction

## Abstract

Pulmonary thrombosis is a fatal complication observed in patients following severe trauma or pulmonary infections. Patients with existing heart failure (HF) are more susceptible to infection-induced lung thrombosis; however, the underlying mechanisms remain poorly understood. This study introduces a severe mouse pulmonary thrombosis model utilizing CD40 knockout (KO) mice following transverse aortic constriction (TAC)-induced HF. While CD40 KO was found to have no detectable effect on left ventricular (LV) structure or function in mice either after TAC or under control conditions, the CD40 KO mice developed profound pulmonary micro-thrombosis after TAC. CD40 deficiency also significantly exacerbated TAC-induced pulmonary leukocyte infiltration (such as total CD45^+^ cells, Mac2^+^ cells and CD3^+^ cells), ∼1.7-fold more pulmonary fibrosis, and pulmonary vessel remodeling, as well as the consequent right ventricular hypertrophy. Mechanistically, our findings indicate that CD40 KO significantly enhanced TAC-induced pulmonary oxidative stress and pulmonary vascular endothelial cell activation, as indicated by upregulated vascular cell adhesion molecule-1 (VCAM-1) and intercellular cell adhesion molecule-1 expression (ICAM-1). Moreover, CD40 KO and TAC synergistically augmented thrombin (49.1 ± 3.0 in CD40 KO TAC group vs 27.2 ± 4.8 in WT TAC group), collagen (51.4 ± 2.5 in CD40 KO TAC group vs 37.8 ± 2.4 in WT TAC group), and ADP-induced platelet aggregation (40.1 ± 2.1 in CD40 KO TAC group vs 31.0 ± 1.8 in WT TAC group) and blood clot contraction(0.92 ± 0.01 in CD40 KO TAC group vs 0.72 ± 0.03 in WT TAC group) in mice. Furthermore, mild but significant pulmonary micro-thrombosis and increased blood clot retraction (0.80 ± 0.02 in CD40 KO sham group vs 0.66 ± 0.02 in WT sham group) were also observed in CD40 KO mice under baseline (control) conditions. Collectively, these results demonstrate that the profound pulmonary micro-thrombosis observed in CD40 KO mice is the outcome of a synergistic effect involving an inherent platelet defect in CD40 KO mice, combined with HF-induced pulmonary endothelial oxidative stress, endothelial activation, and systemic platelet activation. This unique lung micro-thrombosis model may serve as a useful tool for investigating the mechanisms and therapeutic strategies for pulmonary micro-thrombosis, particularly under conditions of existing HF.

## Graphical abstract

**Figure:**
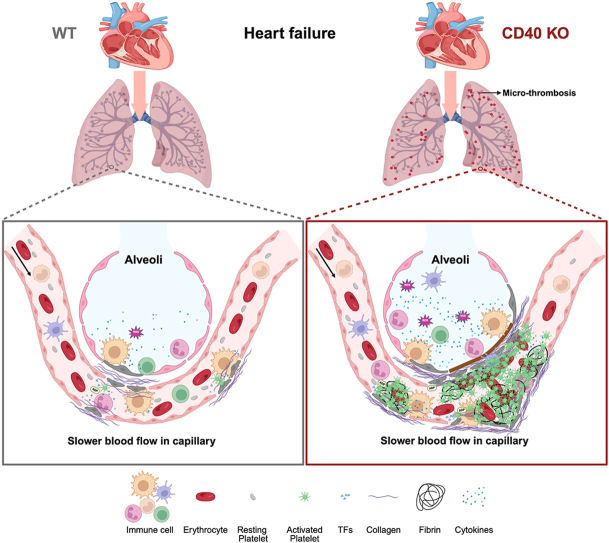

## 1 Introduction

Pulmonary thrombosis is a common and frequently fatal complication in patients with Acute Respiratory Distress Syndrome (**ARDS**), typically following severe lung trauma or infection, such as influenza or Coronavirus Disease 2019 (COVID-19) [[1], [2], [3], [4]].The incidence of pulmonary thrombosis in patients with COVID-19 and seasonal influenza correlates with poor clinical outcomes, particularly among older individuals with preexisting cardiovascular diseases, including hypertension and chronic heart failure (**HF**) [[4], [5], [6], [7]]. While prothrombotic factors can activate platelets (thrombocytes) and fibrin to facilitate the formation of blood clots and vessel injury repair, however, the abnormal platelet activation, microvascular injury, and inflammation also cause pulmonary microvascular thrombosis in the context of ARDS [8].

HF frequently arises from underlying cardiovascular conditions, including myocardial infarction, systolic hypertension, idiopathic cardiomyopathy, myocarditis, and various cardiac defects [9,10]. Patients experiencing chronic left ventricular (LV) failure often progress to WHO Group-2 pulmonary hypertension (PH) and subsequent right ventricular (RV) hypertrophy/failure; this transitional process is generally characterized as HF progression [11,12].

To experimentally induce pressure overload-induced HF, increased LV pressure via transverse aortic constriction (TAC) is a widely utilized method in animal models. Previous investigations by our group and others have consistently demonstrated that TAC-induced HF in mice is associated with severe lung inflammation, vessel remodeling, abnormal energy metabolism, and fibrosis [[13], [14], [15], [16], [17]]. Furthermore, HF patients exhibit heightened vulnerability to respiratory stressors such as air pollution, bacterial or viral infections, and consequent acute respiratory distress syndrome (ARDS) and lung thrombosis [1,2,5,6,18]. Consistent with these clinical observations, we previously demonstrated that air pollution profoundly exacerbates lung inflammation in HF mice [15]. Additionally, inhibition of the inflammatory response was shown to attenuate lung remodeling and HF progression in mice with existing LV dysfunction [14,19,20]. We also found that HF results in increased pulmonary endothelial intercellular cell adhesion molecule 1 (ICAM-1) and vascular cell adhesion molecule 1 (VCAM-1) expression [[13], [14], [15]], suggesting that heightened pulmonary oxidative stress and vascular endothelial cell activation may promote the binding of endothelial cells with immune cells and platelets, thereby facilitating the formation of pulmonary thrombosis. Indeed, HF patients are recognized as being at a higher risk of pulmonary thrombosis [4,7,21].

CD40 is a crucial co-stimulatory protein expressed on antigen-presenting cells (APCs), such as B cells and macrophages, as well as on non-immune cells, including platelets and endothelial cells [[22], [23], [24], [25], [26]]. The interaction between CD40 and its ligand, CD40L (CD154), is not only critical for both cellular and humoral adaptive immunity, but studies also suggest the CD40/CD40L pathway regulates endothelial and platelet activation, as well as thrombosis [[24], [25], [26], [27], [28], [29], [30]]. Given the central role of this pathway in mediating diverse immune and inflammatory responses, therapeutic strategies have mainly focused on either stimulating the CD40/CD40L pathway for cancer treatment or attenuating it to manage autoimmunity or organ transplant rejection [29,31].

To test the hypothesis that inhibition of CD40 signaling might affect heart failure (HF) development and consequent pulmonary remodeling by reducing the cardiovascular inflammatory response, we investigated the effect of CD40 knockout (KO) on transverse aortic constriction (TAC)-induced cardiac inflammation, hypertrophy, and dysfunction in mice. Contrary to expectations, we found that CD40 KO had no detectable effect on TAC-induced left ventricular inflammation, hypertrophy, and dysfunction. Instead, CD40 KO caused severe lung micro-thrombosis and endothelial activation in mice after HF development, indicating a synergistic effect between HF-associated pulmonary endothelial activation and CD40 deficiency in promoting lung micro-thrombosis.

## 2 Materials and methods

**Animals and experimental design:** CD40 KO mice (B6.129P2–*Cd40*^*tm1Kik*^/J; stock NO: 002928) and wild type (WT) C57BL/6J mice were purchased from Jackson Laboratory and Shanghai SLAC Laboratory Animal Co, Ltd. Based on the information provided by Jackson Laboratory, CD40 KO strain has been backcrossed to C57BL/6J mice for at least 10 generations. Male mice 8-10 weeks of age were subjected to TAC created using a 27G needle or sham surgery [13].

All animal procedures were approved by the Institutional Animal Care and Use Committee (IACUC) of Shanghai Tenth People's Hospital of Tongji University, China, and adhered to institutional and national guidelines. Mice were anesthetized using 1% Nembutal (pentobarbital sodium salt) or 1.5-2.5% isoflurane.

**Transverse aortic constriction (TAC):** Briefly, the anesthetized mice were placed in the supine position. A horizontal incision approximately 5 mm in length was made at the level of the suprasternal notch to allow direct access to the transverse aorta while minimizing entry into the pleural space. Aortic constriction was performed by ligating the aorta between the right innominate artery and the left carotid artery over a 27-gauge needle using 5.0 silk suture with the aid of a dissecting microscope. The needle was then quickly removed, which left the calibrated constriction in place. For the sham surgery group, the identical procedure was performed, but without the final ligation step. Echocardiography was performed before surgery, 4 and 8 weeks after TAC using Visualsonics Vevo 2100 system as previously described [13].

**Evaluation of LV hemodynamics:** Mice were continuously anesthetized by 1.5% isoflurane and 95%O_2_, and body temperature was maintained at 37 °C with a heating pad. A 1.2 Fr. pressure catheter (Transonic Systems, Inc., USA) was introduced through the right common carotid artery into the ascending aorta and then advanced into the LV measuring the LV end-systolic pressure (LVESP), end diastolic pressure (LVEDP), heart rate (HR), and maximum/minimum rates of change of LV pressure (dp/dt_max_ and dp/dt_min_) as previously described [13,[32], [33], [34]].

**Sample collection and tissue processing**: Following anesthesia, blood samples were collected from mouse abdominal aorta. These samples were used to quantify p-selectin expression, perform fibrinogen (Fg)-binding and platelet aggregation assays, and conduct clot retraction tests [35]. Cardiac and lung tissues were harvested and weighed. Tissues were subsequently processed for histological and molecular analyses.

**Histological and molecular analysis:** For histological analysis, tissues were fixed in 4% formaldehyde and embedded in paraffin for general histological staining. Lung tissues designated for DHE (dihydroethidium) staining were embedded in Optimal Cutting Temperature (OCT) compound (Sakura Finetek, #4583). Tissues intended for further protein and RNA analysis were flash-frozen immediately in liquid nitrogen and stored at a −80 °C freezer.

**Platelet preparation:** After mice were anesthetized, whole blood was collected from the abdominal aorta into a 5 mL plastic syringe containing White's buffer (pH 6.4). An equal volume of 0.9% NaCl, pre-maintained at 37 °C, was added and mixed gently. To inhibit platelet activation, apyrase and prostaglandin E1 (PGE1) were added.

Platelet-rich plasma (PRP) was obtained via a soft spin centrifugation. The remaining cellular precipitate was subjected to a hard spin to isolate the platelet pellet, which was subsequently washed and resuspended in Tyrode's buffer to produce a washed platelet suspension. Platelet concentration was determined using an automatic animal blood cell analyzer and adjusted using Tyrode's buffer. Prior to functional assays, the washed platelets were incubated at 37 °C for 30 min to 1 h.

**Platelet aggregation assay:** Platelet aggregation was assessed using a CHRONO-LUME aggregometer (Havertown, PA), based on the turbidometric method of Born. Washed platelets were stimulated with the following agonists: adenosine diphosphate (ADP), collagen, and thrombin. Platelet aggregation was determined.

**Clot retraction assay:** Clot retraction was assessed in a platelet aggregation tube. Washed platelets (100 μl) were combined with human plasma (300 μl), and coagulation was initiated by the addition of thrombin (final concentration: 1U/ml). The mixture was incubated at 37 °C. Clot retraction was monitored by capturing digital photographs at indicated time points. The resulting clot size was quantified using ImageJ software, and the retraction ratio was calculated using the formula: [1- (final clot size/initial clot size)]. This methodology allows for the quantitative assessment of platelet function and clot stability.

**Statistics:** Shapiro-Wilk test was used to test normality of data distribution before parametric or non-parametric tests were applied. *Data were presented as mean ± SEM. For comparisons of two groups, unpaired two tailed Student's t-test was used. For one-way nonparametric tests, data were analyzed and evaluated by one way ANOVA followed by Bonferroni post hoc analysis or Kruskal-Wallis's test as appropriate. The null hypothesis was rejected at P < 0.05.*

## 3 Results

**Fig. 1:**
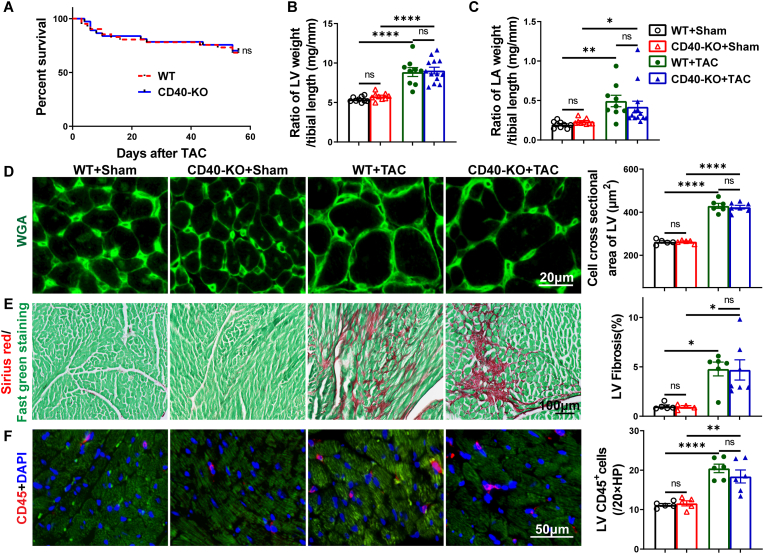
TAC caused similar left ventricular hypertrophy, fibrosis and accumulation of immune cells in WT and CD40 KO mice. Post-TAC survival analysis of WT (n = 41) and CD40 KO (n = 37) mice (A). The ratios of LV and LA weight to tibial length of WT and CD40 KO mice under control or TAC condition (n = 9-13), Sham indicates no actual TAC (B, C). Representative images and summary data for LV myocyte cross-sectional area determined by FITC-conjugated wheat germ agglutinin (WGA) staining (n = 5-7) (D). Representative images and quantitative data of LV fibrosis by Sirius red/Fast green staining (n = 4-7) (E). CD45 immunostaining (red) and quantitative data of LV leukocyte infiltration (n = 5-6) (F). Survival rate was analyzed by Kaplan-Meier method and compared by log-rank test. All quantitative data are reported as means ± SEM. Data were analyzed using one-way ANOVA followed by Bonferroni post hoc analysis. ns indicates nonsignificant (*p* > 0.05),∗*p* < 0.05, ∗∗*p* < 0.01, ∗∗∗*p* < 0.001,∗∗∗∗*p* < 0.0001.

**Fig. 2:**
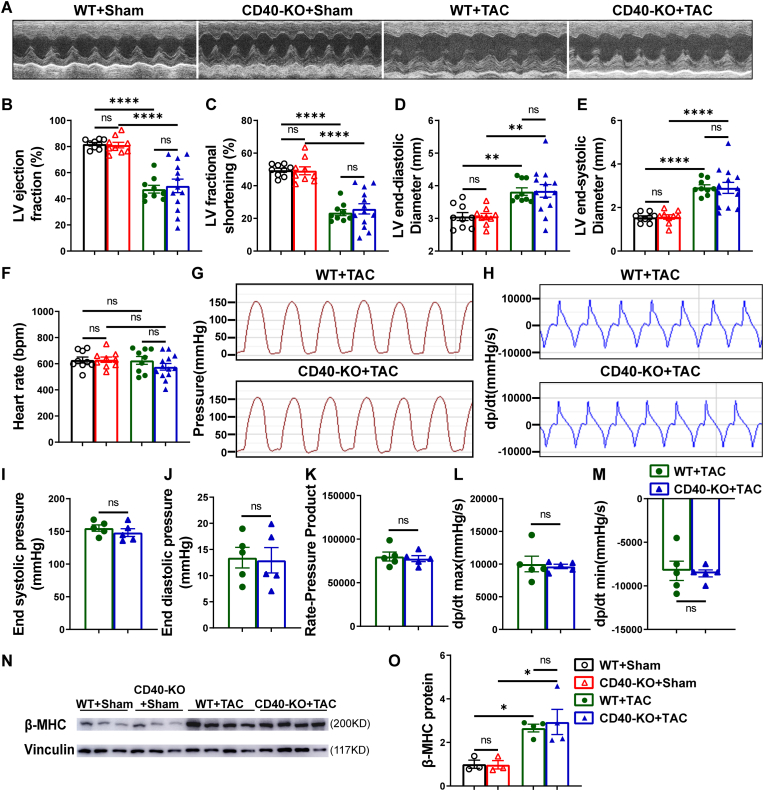
TAC caused comparable LV dysfunction in WT and CD40 KO mice. Representative echocardiograms of each group (A). Summary data for LV ejection fraction (EF%), fractional shortening (FS%), LV end-diastolic and end-systolic diameter (LV-EDD, LV-ESD) and heart rate (n = 9-13) in each group (B–F). LV pressure of WT (n = 5) and CD40 KO (n = 5) mice were measured at 8 weeks after TAC, representative invasive pressure curve(G) with quantifications of end-systolic pressure (ESP)(I) and end-diastolic pressure (EDP)(J). Representative dp/dt curve(H) with quantifications of dp/dtmax (maximal rate of change in systolic pressure over time) (L) and dp/dtmin (minimal rate of change in pressure over time) (M). The rate-pressure product (K) was calculated by multiplying the heart rate by the LV end-systolic pressure. Western blot of β-MHC and vinculin (loading control, n = 3-4) (N, O). All quantitative data are reported as mean ± SEM. The statistical significance was assessed using two-tailed Student's unpaired t tests(I-M). Data were analyzed using one-way ANOVA followed by Bonferroni post hoc analysis (B–F, N, O). ns indicates nonsignificant (*p* > 0.05),∗*p* < 0.05, ∗∗*p* < 0.01, ∗∗∗*p* < 0.001,∗∗∗∗*p* < 0.0001.

**TAC surgery induced comparable levels of left ventricular (LV) inflammation, hypertrophy, and dysfunction in both CD40 KO mice and wild-type (WT) mice.** Under control (sham operation) conditions, CD40 KO mice exhibited cardiac structure and LV function comparable to those of WT mice (Fig. 1B–E; Fig. S1; Table S1; Fig. 2A–F; Fig. S2A and B). To investigate the impact of CD40 deficiency on pressure overload-induced LV inflammation and dysfunction, TAC was performed in both WT and CD40 KO mice. TAC caused similar mortality rates in WT and CD40 KO mice (Fig. 1A). As anticipated, TAC caused significant LV hypertrophy, evidenced by substantial increases in LV weight, left atrial (LA) weight, and their respective ratios normalized to tibial length across both WT and CD40 KO mice (Fig. 1B and C; Table S1). The degree of TAC-induced LV hypertrophy was similar between the WT and CD40 KO groups (Fig. 1B, D,E; Table S1). Histological examination further confirmed that TAC resulted in comparable LV cardiomyocyte hypertrophy, fibrosis, and CD45^+^ leukocyte infiltration in both genotypes (Fig. 1D–F).

Echocardiographic assessments revealed similar significant reductions in LV ejection fraction and fractional shortening at 4- and 8-weeks post-TAC, as well as similar changes in LV end-systolic and end-diastolic dimensions and volumes in WT and CD40 KO mice (Fig. 2A–F; Fig. S2A and B; Fig. S3). Moreover, TAC caused similar changes of LV end-systolic pressure (LVESP), end-diastolic pressure (LVEDP), rate-pressure product, LV dP/dt_max_, and LV dP/dt_min_ in WT mice and CD40 KO mice (Fig. 2G–M). To further assess biochemical markers of LV remodeling, Western blot analyses were conducted to examine overall LV β-myosin heavy chain (β-MHC) expression. CD40 KO had no effect on LV β-MHC protein expression in mice under basal conditions or after TAC as compared with corresponding wild type mice (Fig. 2N and O).

**Fig. 3:**
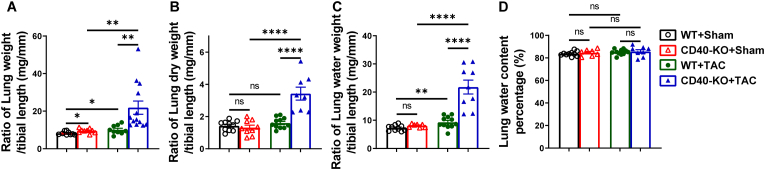
CD40 KO aggravated TAC-induced increase of lung weight. The ratios of lung wet weight, lung dry weight, lung water weight to tibial length (A-C). Lung water content percentage ((lung wet weight – lung dry weight)/lung wet weight × 100%) of each group mice (n = 7-13) (D). All quantitative data are reported as mean ± SEM. Data were analyzed using one-way ANOVA followed by Bonferroni post hoc analysis. ns indicates nonsignificant (*p* > 0.05),∗*p* < 0.05, ∗∗*p* < 0.01, ∗∗∗*p* < 0.001,∗∗∗∗*p* < 0.0001.

**CD40 KO exacerbates TAC-induced pulmonary complications, leading to increased lung weight and pulmonary microvascular thromboses.** HF or LV failure often causes increased lung weight, and the increased lung weight is generally correlated with the degree of LV failure. While TAC caused similar LV dysfunction in wild type and CD40 KO mice, to our surprise, CD40 KO dramatically exacerbated TAC-induced increases of lung weight, and its ratio to tibial length (Fig. 3A; Table S1). To determine whether the increase of lung weight was an outcome of lung edema, we determined the lung water weight, dry weight, and their ratios to tibial length, as well as the percentage of water content in the lung tissues. We found that lung water weight, dry weight, and their ratios to tibial length were increased in CD40 KO mice as compared with WT mice after TAC (Fig. 3B and C). However, the percentage of water content in lung tissues were similar in all experimental groups (Fig. 3D), indicating that lung edema was not responsible for TAC-induced increase of lung weight in CD40 KO mice.

**Fig. 4:**
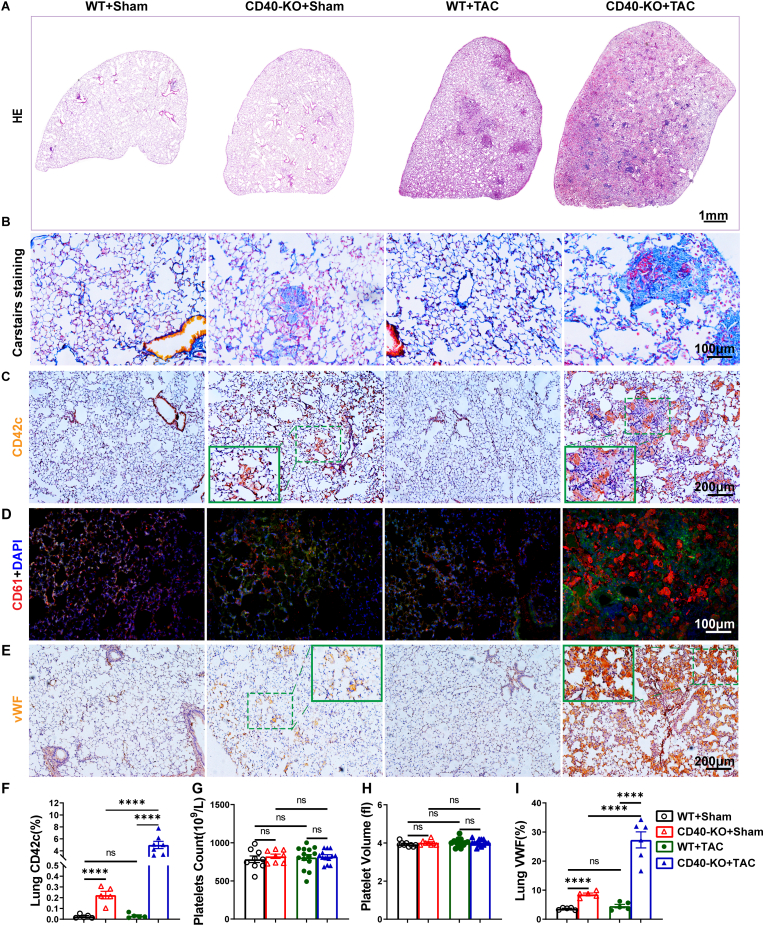
CD40 KO caused robust pulmonary microvascular thrombosis in mice after TAC. Representative images of hematoxylin and eosin (HE) staining of lung tissues from the experimental groups(A)**.** Representative immunostaining images of Carstairs staining of the lung (Fibrin: bright red, platelets: gray blue or navy, collagen: bright blue), CD42c, CD61 and vWF in the lung (B-E). Quantitative data of CD42c immunofluorescence staining in the lung (n = 5-7) (F). The platelet counts in each group of mice (n = 8-14) (G). Mean platelet volume (n = 8-14) (H). Quantitative data of vWF in the lung (n = 5-6) (I). The CD42c and vWF staining were expressed as a percentage of positive staining area to the total area. All quantitative data are reported as mean ± SEM. Data were analyzed using one-way ANOVA followed by Bonferroni post hoc analysis. ns indicates nonsignificant (*p* > 0.05),∗*p* < 0.05, ∗∗*p* < 0.01, ∗∗∗*p* < 0.001,∗∗∗∗*p* < 0.0001.

To determine the cause of lung consolidation in CD40 KO mice, histological staining was performed. Interestingly, hematoxylin and eosin (HE) and Carstairs staining show that TAC caused profound lung vascular thrombosis in CD40 KO mice (Fig. 4A and B; Fig. S4A). Immunobiological staining of platelet markers CD42c and CD61 further confirmed increased platelet aggregation in lung vessels in CD40 KO mice after TAC (Fig. 4C and D; Fig. S4B). In addition, co-staining of vessel smooth muscle and platelet markers showed that most of the aggregated platelets are not in the fully muscularized vessels (Fig. S4C). Interestingly, CD42c^+^ and CD61^+^ positive staining mainly occurred inside small pulmonary vessels and capillaries (micro vessels located inside the alveolar walls), but not in the large fully muscularized pulmonary vessels. CD42c^+^ staining was also observed inside the alveoli (Fig. S5). Overall, these changes indicate that the lung thrombosis is predominantly micro-vascular thrombosis, but bleeding inside alveoli was also noted. While quantified data clearly shows that pulmonary CD42c^+^ area was only mildly but significantly increased in CD40 KO mice under control conditions or the sham conditions, pulmonary CD42c^+^ area was drastically increased in CD40 KO mice after TAC (Fig. 4C–F). Collectively, these data indicate that CD40 KO caused severe pulmonary micro-thrombosis in mice after TAC.

**CD40 KO exacerbated thrombin, collagen, and ADP-induced platelet aggregation in mice after HF.** Since platelets contribute to vessel injury repair and pulmonary thrombosis development, we determined the peripheral blood platelet content in each experimental group. We found that CD40 KO did not affect blood platelet count and platelet volume under both control conditions and after TAC (Fig. 4G and H). In addition, immunostaining showed that expression of von Willebrand Factor (vWF), an important glycoprotein for thrombosis formation, was significantly increased in CD40 KO mice under control conditions, and TAC further increased vWF expression in the thrombotic areas (Fig. 4E–I).

**Fig. 5:**
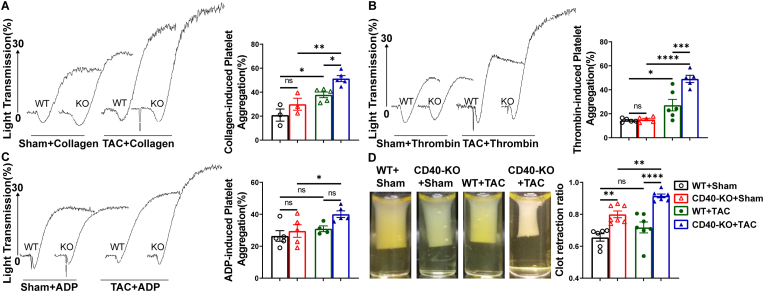
CD40 KO exacerbated platelet aggregation and blood clot retraction in mice after TAC. The aggregation levels of WT and CD40 KO mouse platelets in response to 1.2 μg/ml collagen stimulation (n = 3-5) (A). The aggregation levels of WT and CD40 KO mouse platelets in response to 0.067U/ml thrombin stimulation (n = 5-6) (B). The aggregation levels of WT and CD40 KO mouse platelets in response to 3.4U/ml ADP stimulation (n = 4-5) (C). Clot retraction of WT and CD40 KO mouse platelets (n = 5-7) (D). All quantitative data are reported as mean ± SEM. Data were analyzed using one-way ANOVA followed by Bonferroni post hoc analysis. ns indicates nonsignificant (*p* > 0.05),∗*p* < 0.05, ∗∗*p* < 0.01, ∗∗∗*p* < 0.001,∗∗∗∗*p* < 0.0001.

Given that HF-induced lung remodeling could precipitate the activation of platelet aggregation via the exposure of sub-endothelial collagen in injured vessels, an increase in tissue adenosine diphosphate (ADP) content due to poor perfusion, and heightened thrombin production in injured tissues, we further evaluated platelet aggregation in response to collagen, thrombin, and ADP in both WT and CD40 KO mice. Collagen and thrombin induced significant platelet aggregation in both WT and CD40 KO mice following TAC (Fig. 5A and B); however, collagen and thrombin-induced platelet aggregations were significantly augmented in CD40 KO mice post-TAC compared with WT mice post-TAC (Fig. 5A and B). ADP also caused significantly greater platelet aggregation exclusively in CD40 KO mice after TAC (Fig. 5C). Under control conditions, however, platelet aggregations in response to collagen, thrombin, and ADP were comparable between CD40 KO and WT mice (Fig. 5A–C).

We further assessed platelet granule secretion and integrin αIIbβ3 activation capacity by detecting the mean fluorescence intensity of P-selectin expression and fibrinogen (Fg) binding under resting conditions or after thrombin activation, a process controlled by the so-called inside-out signal. CD40 KO had no detectable effect on platelet P-selectin expression and Fg binding under control conditions (Fig. S6). Thrombin stimulation caused significant increases of P-selectin expression and Fg binding in platelets across all experimental groups, but thrombin-induced maximal P-selectin expression and Fg binding on platelets were similar in all groups (Fig. S6). This suggests that the enhanced platelet aggregation observed after thrombin in CD40 KO mice post-TAC was not attributable to alterations of platelet P-selectin expression or Fg binding capacity.

Since platelet defects and chronic inflammation are often associated with spleen enlargement, we determined spleen weights and their ratios to bodyweight or tibial length in these mice. We found that CD40 KO did not significantly affect spleen weight under control conditions or after TAC (Fig. S7; Table S1).

**CD40 KO significantly exacerbated blood clot retraction in mice after HF.** After platelet aggregation is initiated by fibrinogen binding, thrombin catalyzes crucial coagulation reactions, converting soluble fibrinogen into insoluble fibrin strands. The subsequent fibrin interaction with the platelet cytoskeleton regulates blood clot retraction and thrombus stabilization. To understand the mechanism of increased lung thrombosis in CD40 KO mice, we further determined the blood clot retraction in these mice. Interestingly, blood clot retraction was already significantly increased in control CD40 KO mice (Fig. 5D). TAC tended to increase clot retraction in WT mice, but this increase was not statistically significant (Fig. 5D). However, blood clot retraction was significantly enhanced in CD40 KO mice after TAC compared with corresponding WT mice (Fig. 5D), indicating increased blood clot retraction or thrombus stabilization in CD40 KO mice after HF.

**Fig. 6:**
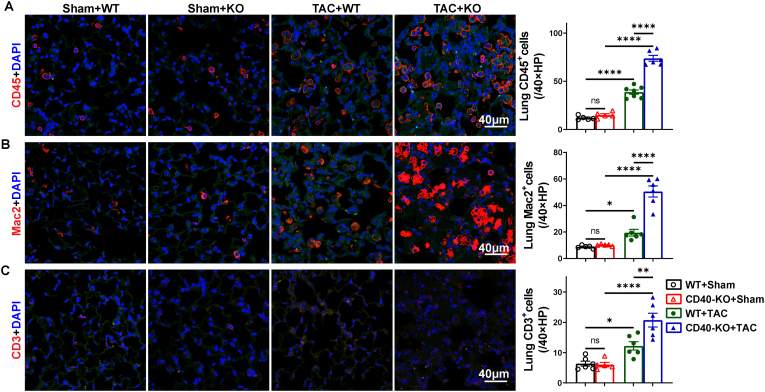
CD40 KO aggravated TAC-induced pulmonary leukocyte infiltration. Representative images and quantitative data of CD45^+^cells, Mac2^+^cells and CD3^+^cells in lungs (n = 5-7) (A-C). All quantitative data are reported as mean ± SEM. Data were analyzed using one-way ANOVA followed by Bonferroni post hoc analysis. ns indicates nonsignificant (*p* > 0.05),∗*p* < 0.05, ∗∗*p* < 0.01, ∗∗∗*p* < 0.001,∗∗∗∗*p* < 0.0001.

**CD40 KO exacerbated TAC-induced lung inflammation in mice.** Since inflammation regulates thrombosis formation, we further examined lung leukocyte infiltration in CD40 KO and WT mice. While TAC caused pulmonary accumulations of CD45^+^ leukocytes, macrophages and CD3^+^ T cells in both wild type and CD40 KO mice, TAC caused significantly greater accumulations of CD45^+^ leukocytes, macrophages and T cells in CD40 KO mice as compared with WT mice (Fig. 6A–C). Furthermore, real-time PCR showed that CD40-KO had no apparent effects on lung interleukin-1beta (IL-1β), IL-6, IL-10, interferon-gamma (IFN-γ) and monocyte chemoattractant protein-1 (MCP-1) under control conditions (Figs. S8A,B,D-F), but CD40 KO significantly reduced the expression of IL-8 and tumor necrosis factor-α (TNFα) under control conditions (Fig. S8C and G). TAC caused significantly greater increases of lung IL-1β, IL-6, IL-10, MCP-1, and TNFα in CD40 KO mice as compared with corresponding WT mice (Figs. S8A, B, D, F, G). TAC resulted in similar increases of lung IFN-γ mRNA in CD40 KO and WT mice (Fig. S8E).

**Fig. 7:**
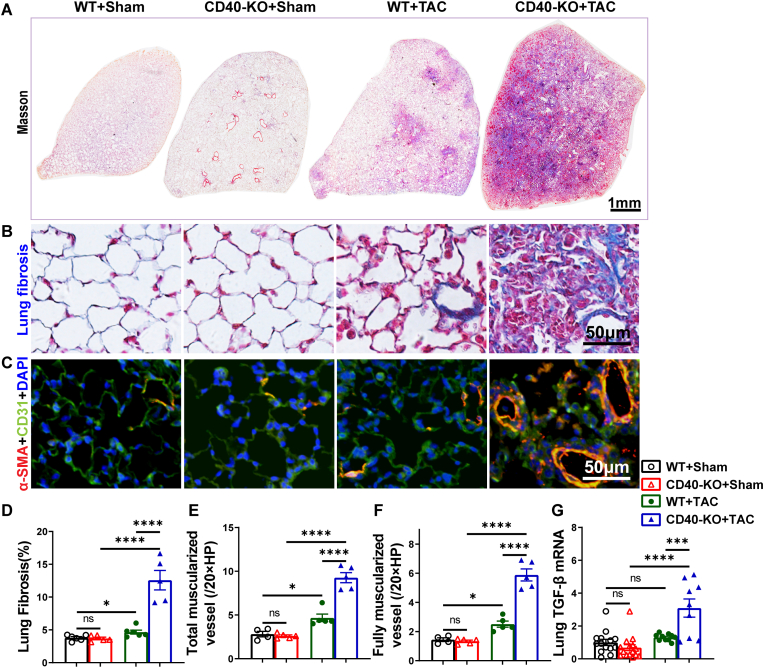
CD40 KO exacerbated pulmonary fibrosis and vessel remodeling in mice after TAC. Representative images (A-C) and quantitative data (D-F) of Masson's trichrome staining and smooth muscle α-actin (red) of the lung tissues. Quantitative RT-PCR result of pulmonary TGFβ (normalized to 18S) (G). All quantitative data are reported as mean ± SEM. Data were analyzed using one-way ANOVA followed by Bonferroni post hoc analysis. ns indicates nonsignificant (*p* > 0.05),∗*p* < 0.05, ∗∗*p* < 0.01, ∗∗∗*p* < 0.001,∗∗∗∗*p* < 0.0001.

**CD40 KO exacerbated TAC-induced pulmonary fibrosis and vessel muscularization in mice.** We evaluated lung fibrosis using Masson's trichrome stain. We observed that CD40 KO did not affect lung fibrosis under control conditions but significantly exacerbated TAC-induced fibrosis (Fig. 7A, B, D).

Lung vessel muscularization is a common adaptive response to increased pulmonary artery pressure. Since both heart failure (HF) and pulmonary thrombosis can cause lung vessel remodeling and pulmonary hypertension in patients, we determined whether increased lung micro-thrombosis in CD40 KO mice was sufficient to induce pulmonary vessel muscularization. While TAC significantly increased the total number of pulmonary muscularized and fully muscularized vessels in both WT and CD40 KO mice, the increases were significantly greater in CD40 KO mice compared with WT mice (Fig. 7C–E, F). TAC also caused a significantly greater increase in lung mRNA content of transforming growth factor-β (TGF-β) in CD40 KO mice compared with WT mice (Fig. 7G).

**Fig. 8:**
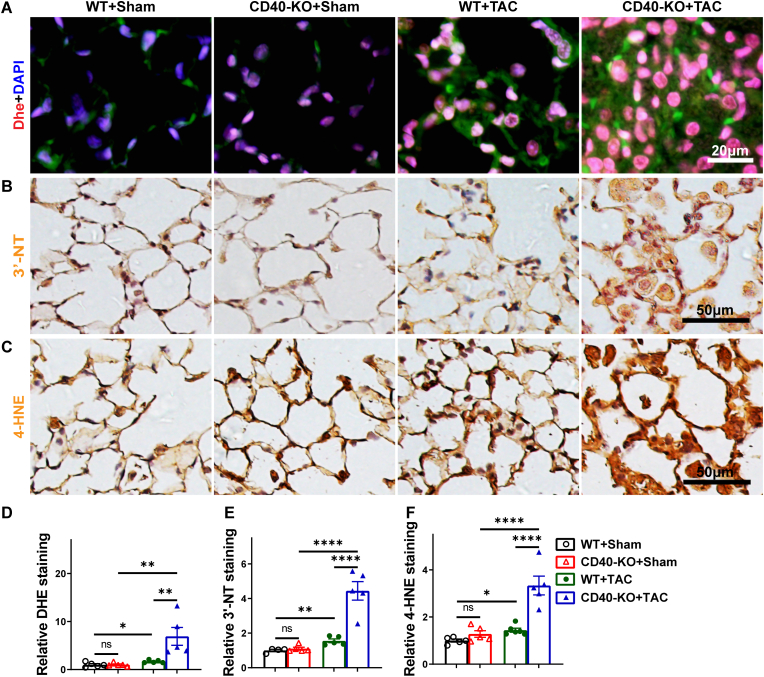
CD40 KO exacerbated pulmonary oxidative stress in mice with existing LV dysfunction. Representative images and Quantitative data of pulmonary dihydroethidium (DHE) staining in mice (n = 5) (A, D), IHC analysis of 3′-nitrotyrosine (B, E) and 4-hydroxynonenal (n = 4-6) (C, F). All quantitative data are reported as mean ± SEM. Data were analyzed using one-way ANOVA followed by Bonferroni post hoc analysis. ns indicates nonsignificant (*p* > 0.05),∗*p* < 0.05, ∗∗*p* < 0.01, ∗∗∗*p* < 0.001,∗∗∗∗*p* < 0.0001.

**CD40 KO aggravated TAC-induced pulmonary oxidative stress in mice.** Oxidative stress plays an important role in the development of HF and other cardiovascular diseases [32,33,36] through inducing initial tissue injury and platelet-dependent thrombosis formation [37]. To investigate whether oxidative stress contributes to the exacerbated lung injury and thrombosis in CD40 KO, we determined the superoxide generation by dihydroethidium (Dhe) staining in lungs. As shown in Fig. 8(A–D), TAC resulted in higher lung superoxide production in both WT and CD40 KO mice, while CD40 KO significantly aggravated the TAC-induced lung reactive oxygen species (ROS) production. In addition, TAC increased the lung 3′-nitrotyrosin(3′-NT) and 4-hydroxynonenal(4-HNE) contents in both wild type and CD40 KO mice, while CD40 KO significantly exacerbated the TAC-induced pulmonary 3′-NT and 4-HNE contents (Fig. 8B, C, E, F).

Adhesion molecules, such as VCAM-1 and ICAM-1 play important roles in promoting leukocyte adhesion to vascular endothelial cells and tissue inflammation. We further detected the pulmonary expression of VCAM-1 and ICAM-1 in these mice. Immunostaining shows that CD40 KO significantly exacerbated TAC-induced pulmonary expression of Vcam1 and Icam1 (Fig. S9).

**Fig. 9:**
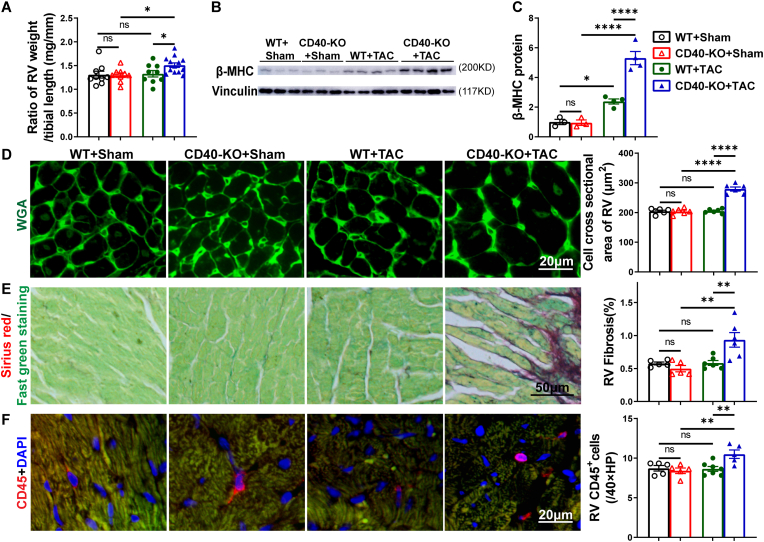
TAC resulted in significant RV inflammation, fibrosis and cardiomyocyte hypertrophy in CD40 KO mice. The ratio of RV weight to tibial length of WT and CD40 KO mice under control or TAC condition (n = 9-13) (A). Western blot of β-MHC and loading control of vinculin (n = 3-4) (B, C)). Representative images and summary data for RV myocyte cross-sectional area determined by FITC-conjugated WGA staining (n = 6) (D). Representative images and quantitative data of RV fibrosis by Sirius red/Fast green staining (n = 5-6) (E). CD45 immunostaining (red) and quantitative data of RV leukocyte infiltration (n = 5-7) (F). All quantitative data are reported as mean ± SEM. Data were analyzed using one-way ANOVA followed by Bonferroni post hoc analysis. ns indicates nonsignificant (*p* > 0.05),∗*p* < 0.05, ∗∗*p* < 0.01, ∗∗∗*p* < 0.001,∗∗∗∗*p* < 0.0001.

**CD40 KO exacerbated TAC-induced RV inflammation, fibrosis, and cardiomyocyte hypertrophy in mice.** As lung thrombosis contributes to lung vessel remodeling, we further determined the RV hypertrophy in WT and CD40 KO mice after TAC. TAC caused significant RV hypertrophy in CD40 KO mice but did not yet cause RV hypertrophy in WT mice as evidenced by increased RV weight and the ratios of RV weight to tibial length (Fig. 9A; Table S1). Western blots showed that TAC caused significant increases of RV β-MHC protein expression in both wild type and CD40 KO mice, but TAC caused a significantly greater increase of RV β-MHC protein expression in CD40 KO mice as compared with wild type mice (Fig. 9B and C). In addition, WGA staining demonstrated that TAC caused significantly more RV cardiomyocyte hypertrophy in CD40 KO mice (Fig. 9D). TAC caused significantly more RV fibrosis and leukocyte infiltration in CD40 KO mice but not in wild type mice (Fig. 9E and F).

## 4 Discussion

The principal finding of this investigation is that CD40 deficiency precipitates profound pulmonary micro-thrombosis in mice with HF induced by TAC, without impacting the trajectory of left ventricular hypertrophy and failure. The observed lung thrombosis in CD40 KO mice is associated with augmented pulmonary leukocyte infiltration, fibrosis, and vascular remodeling, culminating in right ventricular hypertrophy. Mechanistically, CD40 KO significantly enhanced platelet aggregation in response to collagen, thrombin, and ADP, alongside increased blood clot retraction and stabilization post-TAC, independent of platelet count or fibrinogen binding capacity. HF induction alone also resulted in a significant increase in platelet activity following agonist exposure. Furthermore, CD40 KO significantly exacerbated TAC-induced pulmonary oxidative stress, evidenced by markedly elevated lung ROS production, 3′-NT, and 4-HNE levels. Collectively, these results demonstrate that the profound pulmonary micro-thrombosis observed in CD40 KO mice is the outcome of a synergistic effect involving an inherent platelet defect in CD40 KO mice, combined with HF-induced pulmonary endothelial oxidative stress, endothelial activation, and systemic platelet activation.

**The unique post-HF pulmonary microenvironment and subsequent lung remodeling are likely drivers of micro-thrombosis in CD40 KO mice**. HF-induced lung remodeling remains an understudied area of research, and experimental studies specifically investigating HF-induced lung thrombosis are rare. The severity of lung micro-thrombosis detected in CD40 KO mice post-HF development was not fully anticipated.

Thrombosis was exclusively detected within the pulmonary microvasculature; cardiac vessel beds in CD40 KO mice following TAC remained unaffected, as confirmed by CD42c immunofluorescence staining. Minimal micro-thrombosis was also evident in the lungs of sham-operated CD40 KO mice (Fig. 4, Figs. S4 and S5). However, HF is an established risk factor for venous thromboembolism, including lung thrombosis. Existing literature and our prior studies consistently demonstrate that HF induces: (i) massive lung micro-vessel remodeling; (ii) inflammation and fibrosis; (iii) pulmonary hypertension; (iv) pulmonary endothelial cell activation; and (v) increased expression of immune cell adhesion molecules [[13], [14], [15],^38^]. A previous study shown that tissue factors are elevated in microvascular endothelial cells of HF patients [39]. Moreover, elevated soluble CD40L is associated with heart failure and pulmonary hypertension [[40], [41], [42]], and the elevated platelet CD40L expression promotes the interaction between platelets and endothelial cells [40,41], suggesting that CD40/CD40L signaling may contribute to heart failure and HF-induced pulmonary hypertension. Given that inflammation, vessel injury, increased collagen deposition, and hypoxia are established prothrombotic factors that activate platelet aggregation, these HF-induced changes are likely the dominant drivers of thrombus formation in CD40 KO mice post-TAC. In the context of the clinical significance of lung thrombosis in ARDS patients and severe COVID-19 patients with preexisting cardiovascular diseases, additional mechanistic investigations of the pulmonary microvasculature under heart failure conditions are clearly needed.

**The unique pulmonary vasculature and associated hemodynamics likely drive the development of micro-thrombosis in CD40 KO mice**. Specifically, slower lung blood flow is a significant contributor to pulmonary thrombosis. Due to the extensive **lung micro-vessel bed architecture**, the blood flow rate in lung capillaries is inherently slower than in micro-vessels of other vital organs such as the heart and brain. While this slower flow facilitates efficient oxygen and CO_2_ exchanges, it also increases the risk of platelet aggregation under pathological conditions—an essential step for thrombus formation. Given that oxidative stress contributes to vessel injury and platelet activation [37], our findings indicate that increased oxidative stress plays a critical role in lung thrombosis and remodeling in CD40 KO mice post-TAC. Furthermore, pulmonary alveoli and their internal micro-vessels are vulnerable to stress and injury, specifically lacking the robust protection provided by surrounding tissues. Increased pulmonary venous pressure resulting from LV failure can further cause significant mechanical stress and subsequent injury to the capillary walls. Unfortunately, these unique pulmonary mechanical and hemodynamic features represent an ideal environment for the development of micro-thrombosis and vessel injury following the onset of HF.

**The synergetic effect of HF-induced pulmonary remodeling and platelet defection in CD40 KO mice contributes to the profound pulmonary micro-thrombosis**. Since TAC caused similar LV hypertrophy and dysfunction in both WT and CD40 KO mice, the severe pulmonary micro-thrombosis observed in CD40 KO mice post-TAC (but not under control conditions) further supports the notion that HF-induced lung remodeling, specific to the CD40 KO phenotype, was required for the development of robust micro-thrombosis in these mice. The comparable increase in LV inflammation, hypertrophy, fibrosis, and dysfunction between WT and CD40 KO mice following TAC also indicates that CD40 had no significant effect on these specific TAC-induced LV pathologies.

The occurrence of mild pulmonary micro-thrombosis in sham-operated CD40 KO mice suggests that CD40 deficiency causes intrinsic platelet changes, even under control conditions. Concurrently, the significant increases in pulmonary von Willebrand factor (vWF) indicate that an abnormal platelet and endothelial interaction contribute to the enhanced pulmonary thrombosis observed in CD40 KO mice. Moreover, compared with platelets isolated from control animals, platelets obtained from both HF CD40-KO and HF WT mice exhibited heightened sensitivity to thrombin, collagen, and/or ADP-induced platelet aggregation, as well as increased thrombin-induced blood clot contraction. These findings indicate that HF not only induced lung remodeling but also caused significant platelet functional changes in both WT and CD40 KO mice.

The increased platelet activity in HF mice aligns with clinical findings that human HF patients exhibit abnormal platelet structure and function, enhanced platelet activity and auto-aggregation [43,44], and an increased incidence of venous or pulmonary thrombosis [7,21]. This heightened platelet activity and auto-activation following HF might be attributed to elevated cytosolic free calcium concentrations (driven by enhanced sympathoadrenal activation and catecholamine release), inflammation, and ischemia. Increased endothelial injury and expression of tissue factors also contribute to enhanced platelet aggregation and thrombus formation in HF patients [7,21,45]. Moreover, additional systemic and cardiopulmonary changes (such as the increased incidence of cardiac arrythmia, alteration of central circadian clock, and the increase calcium and/or sodium channel activation), may also promote pulmonary thrombosis at least partially enhancing HF development [[46], [47], [48]]. Thus, exacerbated platelet aggregation in response to prothrombotic factors in the CD40 KO mice after the induction of HF was likely another critical factor in promoting the robust development of pulmonary micro-thrombosis in this specific genotype.

**CD40/CD40L pathway effects on inflammatory diseases and thrombosis**. The critical role of the CD40/CD40L pathway in both humoral and cellular immunity has made it an intense target for therapeutic intervention in diseases such as various cancers and autoimmune disorders, including multiple sclerosis (NCT04879628), systemic lupus erythematosus (SLE) (NCT02804763), primary Sjögren's syndrome (NCT04572841), and adult-onset rheumatoid arthritis (NCT02780388) [27,30,49]. However, conflicting evidence exists within the literature. Some patients and animal models have developed thromboembolic complications following the inhibition of CD40 [30,50,51], suggesting that the role of CD40 in thrombosis development may be contingent upon the specific disease context or tissue type. Prior experimental studies have demonstrated that CD40/CD40L signaling promotes thrombosis by enhancing local inflammatory responses and/or activating platelets [7,[24], [25], [26],[52], [53], [54], [55]]. Conversely, other studies have reported thromboembolic complications in experimental animals after inhibition of CD40/CD40L signaling via monoclonal antibodies against CD40L [51,56]. Furthermore, at least one clinical trial targeting the CD40/CD40L pathway was terminated early due to concerns regarding thromboembolic events [49]. Consequently, the potential roles of CD40/CD40L blockade in promoting unwanted thromboembolic complications or infection remain a concern within the field [30,49,51]. Prior investigations have established that CD40/CD40L signaling suppresses endothelial eNOS expression and subsequent nitric oxide (NO) production; conversely, it promotes iNOS expression and NO synthesis within immune cells [57,58]. This pathway further serves as a critical nexus between systemic inflammation and haemostasis, typically upregulating iNOS and enhancing thrombotic stability [59,60]. Clinical evidence supports this pathogenic role, as a recent study indicated that neutralizing soluble CD40L reduced major cardiovascular events in high-flux hemodialysis patients [61]. While the prevailing literature identifies CD40/CD40L signaling as pro-inflammatory and pro-thrombotic, our current observation—that CD40 KO significantly exacerbated TAC-induced pulmonary thrombosis—presents a counterintuitive finding that warrants further mechanistic exploration.

Nevertheless, most clinical trials indicate that anti-CD40 or anti-CD40L monoclonal antibodies are generally safe and well tolerated, with no evidence of increased thromboembolic events [49,62]. Experimental studies generally suggest that inhibition of CD40 or CD40L by genetic deficiency or blocking antibodies is effective in attenuating inflammation and thrombosis development [30,49,[53], [54], [55]]. However, as noted, conflicting evidence persists where some patients and animal models have developed complications following inhibition of CD40 [50,51], underscoring that the role of CD40 in thrombosis may be context-dependent. The opposite thromboembolic phenotypes observed after the genetic or pharmacological inhibition of CD40/CD40L in experimental animals warrant additional future investigations, particularly within the framework of preexisting disease models.

**Study limitations**: The present study has several limitations that warrant consideration. First, the mouse TAC-induced HF model is relatively acute compared with the chronic course observed clinically in patients with aortic stenosis or hypertension-induced HF, potentially limiting its full mimicry of human conditions. Second, utilizing a global CD40 KO mouse strain means observed phenotypes may partially reflect chronic adaptation to gene deletion; nevertheless, the resulting lung micro-thrombosis model offers a valuable preclinical platform for testing therapies aimed at pulmonary micro-thrombosis. Third, to minimize confounding stress responses in the animals, we did not perform invasive ventilation or RV pressure measurements; instead, the impact on pulmonary pressure was objectively estimated via correlations with lung vessel muscularization, inflammation, and RV hypertrophy. Fourth, we acknowledge that HF-induced lung inflammation and remodeling are modulated by the degree of LV failure; while highly effective antioxidants are probable attenuators of TAC-induced cardiopulmonary oxidative stress and subsequent pulmonary remodeling in CD40 KO mice, we currently did not experimentally confirm this anticipated protective effect. Fifth, the *ex vivo* nature of our platelet aggregation assays may not perfectly replicate *in vivo* pathological conditions. Finally, we did not quantify cardiac diastolic dysfunction in this study. Finally, our findings demonstrate that multiple factors (such as platelet defects, heart failure-induced pulmonary inflammation, and oxidative stress) clearly underpin pulmonary endothelial injury and micro thrombosis in CD40 KO mice, the precise relative contributions and mechanistic interplay between platelet defects, oxidative stress, and immune cell recruitment remain to be fully elucidated under pathological conditions. A notable limitation of the present study is that it did not definitively establish the specific roles of oxidative stress versus inflammation in the regulation of pulmonary thrombosis within CD40 KO models following HF development.

**Summary**: We demonstrated that TAC causes profound lung micro-thrombosis in CD40 KO mice, which was associated with increased lung inflammation, fibrosis, vessel muscularization, and RV hypertrophy. The lung thrombosis in CD40 KO mice after HF is likely the synergistic effect of the unique microenvironment in HF-induced lung remodeling such as increased oxidative stress and vessel injury, HF-induced platelet modulation, and the platelet defect in CD40 KO mice. In the context that pulmonary thrombosis is one of the major complications in patients with acute respiratory distress syndrome after severe COVID-19 and the major concerns for new drug development, this model may be useful in the study of lung thrombosis in conditions with pre-existing cardiovascular diseases.

## Ethical approval

All animal study were approved by the Institutional Animal Care and Use Committee at Shanghai Tenth People's Hospital of Tongji University, Shanghai, China. This study does not contain clinical studies or patients' data.

## Declaration of generative AI and AI-assisted technologies in the manuscript preparation

During the revision of this manuscript, the author(s) used Google AI Mode searching references and editing some of the paragraphs. After using Google AI Mode, the author(s) reviewed and edited the content as needed and take(s) full responsibility for the content of the published article.

## Funding

This study was supported by grants 81600308 and 91739114 from the 10.13039/501100001809National Natural Science Foundation of China, and a grant 2019M661638 from 10.13039/501100002858China Postdoctoral Science Foundation. The research activities of Chen's Laboratory are currently supported by R01HL161085 and R01HL139797 from NIH, USA.

## CRediT authorship contribution statement

**Wenhui Yue:** Data curation, Formal analysis, Methodology, Writing – original draft, Writing – review & editing. **Yanyan Xu:** Data curation. **Xinyu Weng:** Data curation. **Dongzhi Wang:** Data curation. **Linlin Shang:** Data curation. **Haojie Jiang:** Data curation. **Edward Kenneth Weir:** Writing – review & editing. **Junling Liu:** Methodology, Supervision. **Yawei Xu:** Funding acquisition, Methodology. **Wenliang Che:** Methodology, Writing – review & editing. **Yingjie Chen:** Conceptualization, Supervision, Writing – original draft, Writing – review & editing.

## Declaration of competing interest

The authors declare that they have no known competing financial interests or personal relationships that could have appeared to influence the work reported in this paper.

## Data Availability

Data will be made available on request.

## Abbreviations

HF, heart failure; KO, knockout; TAC, transverse aortic constriction; ARDS, acute respiratory distress syndrome; COVID‐19, coronavirus disease-2019; LV, left ventricular; PH, pulmonary hypertension; RV, right ventricular; ICAM-1, intercellular cell adhesion molecule-1; VCAM-1, vascular cell adhesion molecule-1; APCs, antigen-presenting cells; LA, left atrial; β-MHC, β-Myosin heavy chain; HE, hematoxylin and eosin; vWF, von Willebrand Factor; ADP, adenosine diphosphate; IL, interleukin; IFN-γ, Interferon-gamma; MCP-1, monocyte chemoattractant protein-1; TNFα, tumor Necrosis Factor-α; TGF-β, transforming growth factor-β; Dhe, dihydroethidium; 3′-NT, 3′-nitrotyrosin; 4-HNE, 4-hydroxynonenal.
