# Supplementary material for: Systolic pressure overload caused pulmonary oxidative stress, vessel remodeling and severe microvascular thrombosis in CD40 knockout mice through promoting platelet aggregation
Source: Redox Biol. 2026 Mar 21;92:104122. doi: 10.1016/j.redox.2026.104122 (PMC13054292; doi:10.1016/j.redox.2026.104122)
Supplement: Multimedia component 1 [file mmc1.docx]

**SUPPLEMENTAL MATERIAL**

Systolic pressure overload caused pulmonary oxidative stress, vessel remodeling and severe microvascular thrombosis in CD40 knockout mice through promoting platelet aggregation

Wenhui Yue^1#^, Yanyan Xu^2,3^, Xinyu Weng^1,4,5^, Dongzhi Wang^1,6^, Linlin Shang^1^, Haojie Jiang^7^, Edward Kenneth Weir^5^, Junling Liu^7^, Yawei Xu^1^, Wenliang Che^1#^, Yingjie Chen^5,6#^

Running title: Heart failure induced lung thrombosis

^1^Department of Cardiology, Shanghai Tenth People’s Hospital, Tongji University School of Medicine, Shanghai, 200072, China

^2^Ruijin Hospital, Department of Laboratory Medicine, Shanghai Jiao Tong University School of Medicine, Shanghai, 200025, China

^3^College of Health Sciences and Technology, Key Laboratory of Cell Differentiation and Apoptosis of Chinese Ministry of Education, Shanghai jiao Tong University School of Medicine, Shanghai, 200025, China

^4^Department of Cardiology, Shanghai Institute of Cardiovascular Diseases, Zhongshan Hospital, Fudan University, Shanghai, 200025, China

^5^Lillehei Heart Institute and Department of Medicine, University of Minnesota Medical School, Minneapolis, MN 55455, USA

^6^Department of Physiology & Biophysics, University of Mississippi Medical Center, Jackson, MS 39216, USA

^7^Department of Biochemistry and Molecular Cell Biology, Shanghai Jiao Tong University School of Medicine, Shanghai, 200025, China

**Address for correspondence:**

Wenhui Yue: [yuewenhui3@163.com](mailto:yuewenhui3@163.com) or Wenliang Che: chewenliang@tongji.edu.cn or Yingjie Chen: [ychen2@umc.edu](mailto:ychen2@umc.edu)

**Supplementary Materials and Methods**

**Animals and Study approval:** During the entire study, the mice were fed commercial mouse chow and distilled water ad libitum and were housed under controlled temperatures (22 ± 2 °C) and relative humidity (40–60%) conditions, with a 12-hour light/dark cycle. All studies in experimental animals were approved by the Institutional Animal Care and Use Committee at Shanghai Tenth People's Hospital of Tongji University, China. The ethics certificate reference number is SHDSYY-2021-6143-2.

**Key reagents:** ADP (20398-34-9), apyrase(A6535), and prostaglandin E1 (PGE1, P5515) were purchased from Sigma-Aldrich (St. Louis, MO). Collagen was obtained from Chrono-log (P/N 385, Havertown, PA). α-thrombin was from Enzyme Research Laboratories (HT1002a, South Bend, IN). FITC-conjugated anti-mouse P-selectin antibody was purchased from Becton Dickinson Biosciences (San Jose, CA). Antibody against β-myosin heavy chain (β-MHC), 3’-Nitrotyrosine and 4-Hydroxynonenal were purchased from Abcam PLC (Cambridge, MA). Antibodies against α-smooth muscle actin and vinculin were from Santa Cruz Biotechnology (Dallas, TX). Anti-CD45 antibody was from R&D systems (Minneapolis, MN). Anti-Mac2 antibody was from Cedarlane Laboratories (Burlington, NC). Anti-CD3 antibody was obtained from Genetex (Irvine, CA). Anti-CD42c antibody was from LifeSpan BioSciences Inc. (Seattle, WA). Anti-CD61 antibody was from Novus Biologicals (Littleton, CO). Anti-vWF antibody was from Dako. Sirius red and Fast green Stain Kit was from Chondrex (9046, Redmond, WA). Alexa 647-conjugated Fg was from Life Technologies (Gaithersburg, MD). Summary of used primary antibodies were shown in Table S3.

**P-selectin expression and Fg-binding assay:** Washed platelets resting or activated with 0.01U/mL α-thrombin were incubated with FITC-conjugated P-selectin and Alexa 674-conjugated Fg binding for 20 minutes at room temperature, and then analyzed by flow cytometer[[1](#_ENREF_2)].

**Histopathology staining:** Heart or lung sections (10μm) were stained with Sirius red/Fast green stain kit or Masson trichrome stain kit to assess fibrosis. The sections (5μm) were used for immunofluorescence and immunohistochemical staining. Sections were de-paraffinized and rehydrated. Hematoxylin-eosin staining, Carstairs staining (DG5396, GenMed scientifics Inc.), Masson staining and Sirius red and Fast green staining were performed using a standard method or the protocols of these kits. The Wheat Germ Agglutinin staining was directly performed after rehydrated. The sections used to the antibody staining were treated with Sodium citrate (PH6.0) or Tris-EDTA (PH9.0) through heat-induced epitope retrieval. Using 0.3% H_2_O_2_ to inactivate endogenous peroxidase for the HRP-DAB detection. Sections were blocked by 1% BSA or the diluted serum corresponding the host species of secondary antibody. 4’, 6’-diamidino-2-phenylindole (DAPI) was from Southern Biotech (0100-20). Immunofluorescence of antibody staining was visualized by using a secondary Alexa Fluor 549-conjugated antibody (Jackson ImmunoResearch Laboratories, Inc.). All the sections were examined using a microscope: IX83 Olympus or LSM710 ZEISS. The images were quantified by ImageJ software (NIH). Immunohistochemical or immunofluorescence staining were statistically analyzed through positive cell count, positive area ratio or Integrated Density.

**Western blotting:** The protein was extracted from the LV or RV using RIPA buffer (CST,9806) and 1× protease inhibitor cocktail (Roche, 4693116001), thoroughly lysed and centrifuged at 4°C. Proteins were separated by 10% SDS-PAGE and transferred onto Polyvinylidene difluoride membranes. The membranes were then blocked with 5% non-fat milk solution and incubated with primary antibodies at 4°C, followed by incubation with horseradish peroxidase-conjugated secondary antibody. The information of primary antibodies was presented in Table S3. Images captured using a western blot imaging system (Amersham Imager 600) were analyzed analyzed by ImageJ software.

**Quantitative real-time PCR:** Total RNA of lungs was extracted using TRIzol reagent (Invitrogen,15596018CN) followed by chloroform extraction, and 2μg of total RNA was used for each reverse transcription reaction using a PrimeScript RT Reagent Kit (TaKaRa, RR037Q) followed by quantitative PCR using KAPA SYBR FAST Universal Kit (Kapa biosystems, KR0389) according to the manufacturer’s instructions. Target gene expression was normalized to 18S and estimated by 2^-ΔΔCT^ method. Primer pairs used for corresponding PCR are listed in Table S2.

**Graphical abstract:** Graphical abstract was created in BioRender. Yue, W. (2026) https://BioRender.com/5pe5hqi.

**Supplemental References**

1. Xu Y, Ouyang X, Yan L, Zhang M, Hu Z, Gu J, Fan X, Zhang L, Zhang J, Xue S, Chen G, Su B and Liu J. Sin1 (Stress-Activated Protein Kinase-Interacting Protein) Regulates Ischemia-Induced Microthrombosis Through Integrin alphaIIbbeta3-Mediated Outside-In Signaling and Hypoxia Responses in Platelets. *Arteriosclerosis, thrombosis, and vascular biology*. 2018;38:2793-2805. <https://doi.org/10.1161/ATVBAHA.118.311822>

**Table S1 Anatomic data for WT and CD40 KO mice under control and TAC conditions**

| **Parameters** | **WT+Sham** | **KO+Sham** | **WT+TAC** | **KO+TAC** |
| --- | --- | --- | --- | --- |
| Number of mice | 9 | 9 | 9 | 13 |
| Bodyweight(g) | 26.6±0.62 | 27.6±0.680 | 27.9±0.621 | 27.1±0.668 |
| Left ventricular (LV) weight (mg) | 95.1±2.37 | 100±2.87 | 156±9.50* | 159±7.78* |
| Left atria (LA) weight (mg) | 3.42±0.23 | 4.06±0.220 | 8.72±1.27* | 7.42±1.31* |
| Lung mass (mg) | 145±5.8 | 165±6.41† | 178±14.0* | 385±62.9*† |
| Right ventricular (RV) weight (mg) | 23.1±1.2 | 22.5±0.872 | 23.4±1.19 | 26.5±0.86* |
| Spleen weight(mg) | 76.6±3.4 | 79.9±4.27 | 87.5±4.75 | 79.0±3.51 |
| Ratio of LV weight to bodyweight (mg/g) | 3.58±0.09 | 3.63±0.080 | 5.63±0.387* | 5.87±0.28* |
| Ratio of LA weight to body weight (mg/g) | 0.129±0.01 | 0.147±0.007 | 0.310±0.04* | 0.272±0.05* |
| Ratio of lung weight to body weight (mg/g) | 5.43±0.16 | 6.00±0.277 | 6.69±0.501 | 14.3±2.55*† |
| Ratio of RV weight to body weight (mg/g) | 0.869±0.05 | 0.817±0.027 | 0.841±0.042 | 0.984±0.04*† |
| Ratio of Spleen weight to body weight (mg/g) | 2.88±0.126 | 2.90±0.150 | 3.14±0.154 | 2.92±0.15 |
| Tibial length(mm) | 17.6±0.15 | 17.3±0.210 | 17.6±0.113 | 17.6±0.09 |
| Ratio of LV weight to tibial length(mg/mm) | 5.39±0.11 | 5.77±0.156 | 8.85±0.56* | 9.05±0.42* |
| Ratio of LA weight to tibial length(mg/mm) | 0.194±0.01 | 0.234±0.012 | 0.494±0.07* | 0.042±0.07* |
| Ratio of Lung weight to tibial length(mg/mm) | 8.20±0.308 | 9.52±0.398† | 10.1±0.79* | 21.9±3.52*† |
| Ratio of RV weight to tibial length(mg/mm) | 1.31±0.072 | 1.30±0.048 | 1.33±0.067 | 1.51±0.05*† |
| Ratio of Spleen weight to tibial length(mg/mm) | 4.35±0.200 | 4.61±0.232 | 4.95±0.25 | 4.50±0.19 |

*p<0.05 as compared with corresponding control conditions; † p<0.05 as compared with WT mice under the same condition (Sham or TAC).

**Table S2 Primers used in quantitative real-time PCR**

| Mouse Gene |  |  |
| --- | --- | --- |
| IL-1β | sense | 5’-TCCTGTGTAATGAAAGACGGC-3’ |
|  | antisense | 5’-ACTCCACTTTGCTCTTGACTTC-3’ |
| IL-6 | sense | 5’-ACCAGAGGAAATTTTCAATAGGC-3’ |
|  | antisense | 5’-TGATGCACTTGCAGAAAACA-3’ |
| IL-8 | sense | 5’-ACCTAGGCATCTTCGTCCGT-3’ |
|  | antisense | 5’-GCCAACAGTAGCCTTCACCC-3’ |
| IL-10 | sense | 5’-CTGCTATGCTGCCTGCTCTTACTG-3’ |
|  | antisense | 5’-ATGTGGCTCTGGCCGACTGG-3’ |
| IFN-γ | sense | 5’-TGCTGATGGGAGGAGATGTCT-3’ |
|  | antisense | 5’-TTTCTTTCAGGGACAGCCTGTT-3’ |
| MCP-1 | sense | 5’-TTTTTGTCACCAAGCTCAAGAG-3’ |
|  | antisense | 5’-TTCTGATCTCATTTGGTTCCGA-3’ |
| TNF-α | sense | 5’-ATGTCTCAGCCTCTTCTCATTC-3’ |
|  | antisense | 5’-GCTTGTCACTCGAATTTTGAGA -3’ |
| TGF-β | sense | 5’-CCTGAGTGGCTGTCTTTTGA-3’ |
|  | antisense | 5’-CGTGGAGTTTGTTATCTTTGCTG-3’ |
| Vcam-1 | sense | 5’-ATCTCAGGTGGCTGCACAAGTTG-3’ |
|  | antisense | 5’-AGCGCACAGGTAAGAGTGTTCATC-3’ |
| 18S | sense | 5’-GCAATTATTCCCCATGAACG-3’ |
|  | antisense | 5’-GGCCTCACTAAACCATCCAA-3’ |

**Table S3 Primary antibodies used in the study**

| **Antibody** | **Catalog number** | **Name of the company** |
| --- | --- | --- |
| 3’-NT | ab61392 | Abcam PLC |
| 4-HNE | ab46545 | Abcam PLC |
| Alexa Fluor^TM^ 647- Fibrinogen | F35200 | Invitrogen (Life Technologies) |
| ANP | T-4010 | Peninsula Laboratories, LLC |
| α-SMA | sc-32251 | Santa Cruz Biotechology, Inc |
| β-MHC | Ab11083 | Abcam PLC |
| Biotin-CD42c | LS-C419893 | LifeSpan BioSciences, Inc |
| CD3 | GTX16669 | GeneTex, Inc |
| CD45 | AF114 | R&D systems |
| CD31 | MAB1398Z | Merck Millipore |
| CD61 | NB600-1342 | Novus Biologicals |
| FITC-P-selectin | 553744 | Becton Dickinson Biosciences |
| FITC-WGA | L4895 | Sigma-Aldrich |
| Mac2 | CL8942AP | Cedarlane Laboratories |
| Vinculin | sc-73614 | Santa Cruz Biotechology, Inc |
| vWF | A0082 | Dako |

**
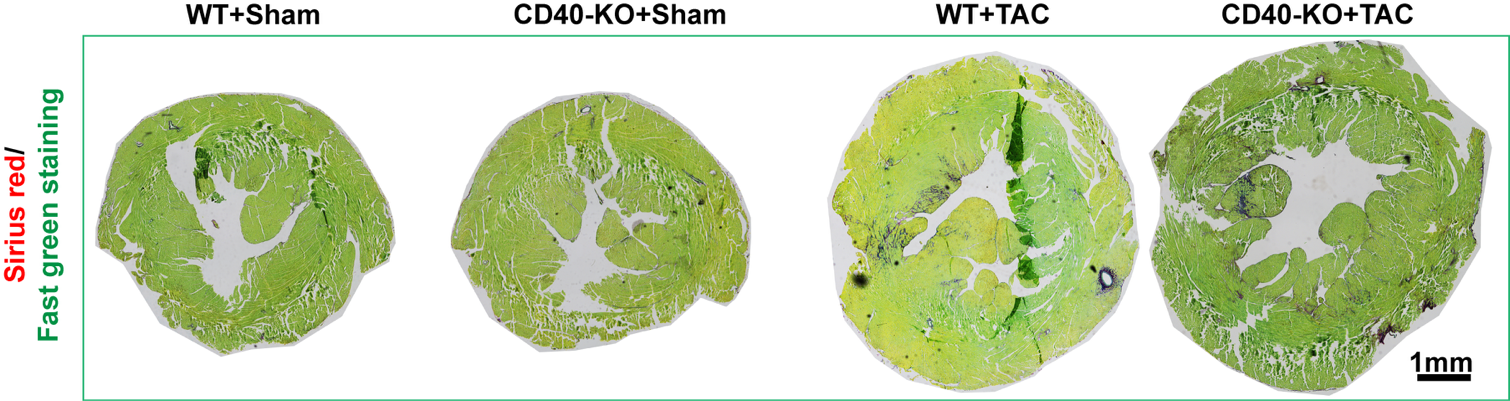
Fig.S1 Representative Fast green staining of LV cross sections showed that TAC caused increase of LV sizes in WT and CD40 KO mice.**

**
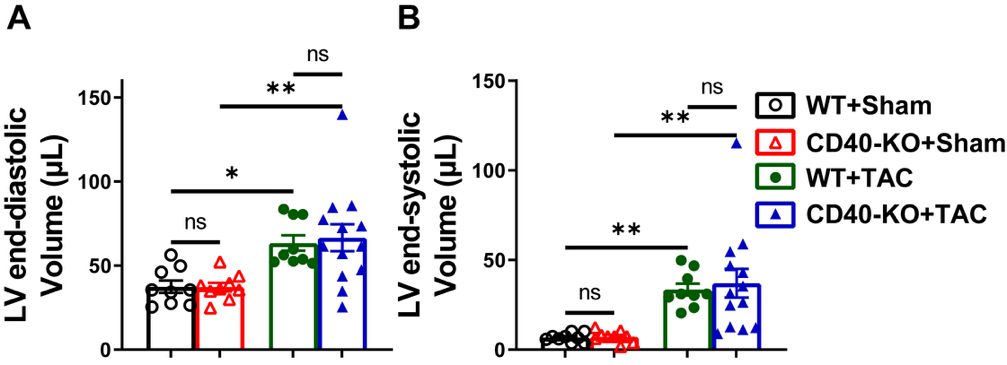
**

**Fig.S2 LV end-diastolic volumes in WT and CD40 KO mice under control conditions or after TAC.** Echocardiographic data for LV end diastolic and systolic volume are shown (n = 9-13) (A, B). All quantitative data are reported as mean ± SEM. Data were analyzed using one-way ANOVA followed by Bonferroni post hoc analysis (A, B). ns indicates nonsignificant (*p*﹥0.05),**p*﹤0.05, ***p*﹤0.01.


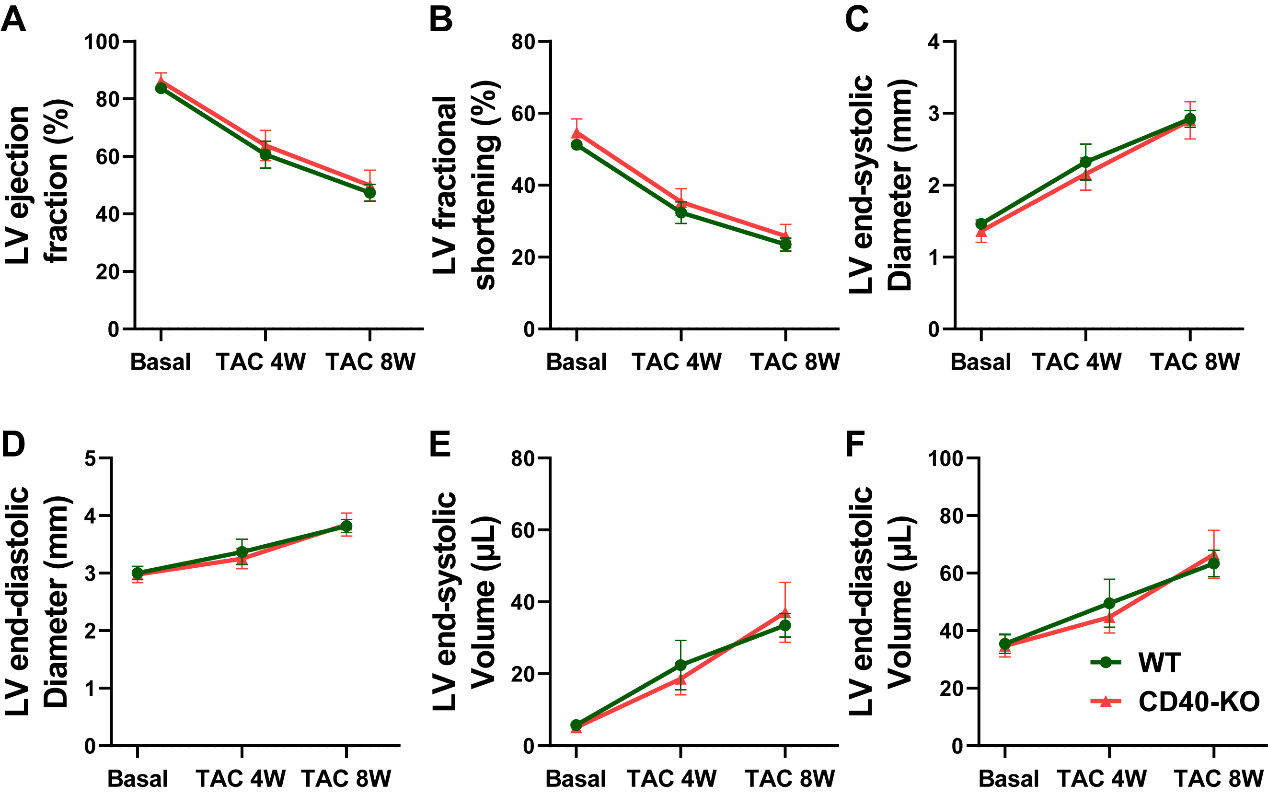


**Fig.S3 Echocardiograph test showed that LV function and dimensions are similar in WT and CD40 KO mice under control conditions, and 4 or 8 weeks after TAC.** Data were collected from mice these under sham condition and at 4/8 weeks after TAC. Summary data for LV ejection fraction (EF%), fractional shortening (FS%), LV end-diastolic and end-systolic diameter (LV-EDD, LV-ESD), LV end diastolic and systolic volume (n=4-13) in each group (A-F).


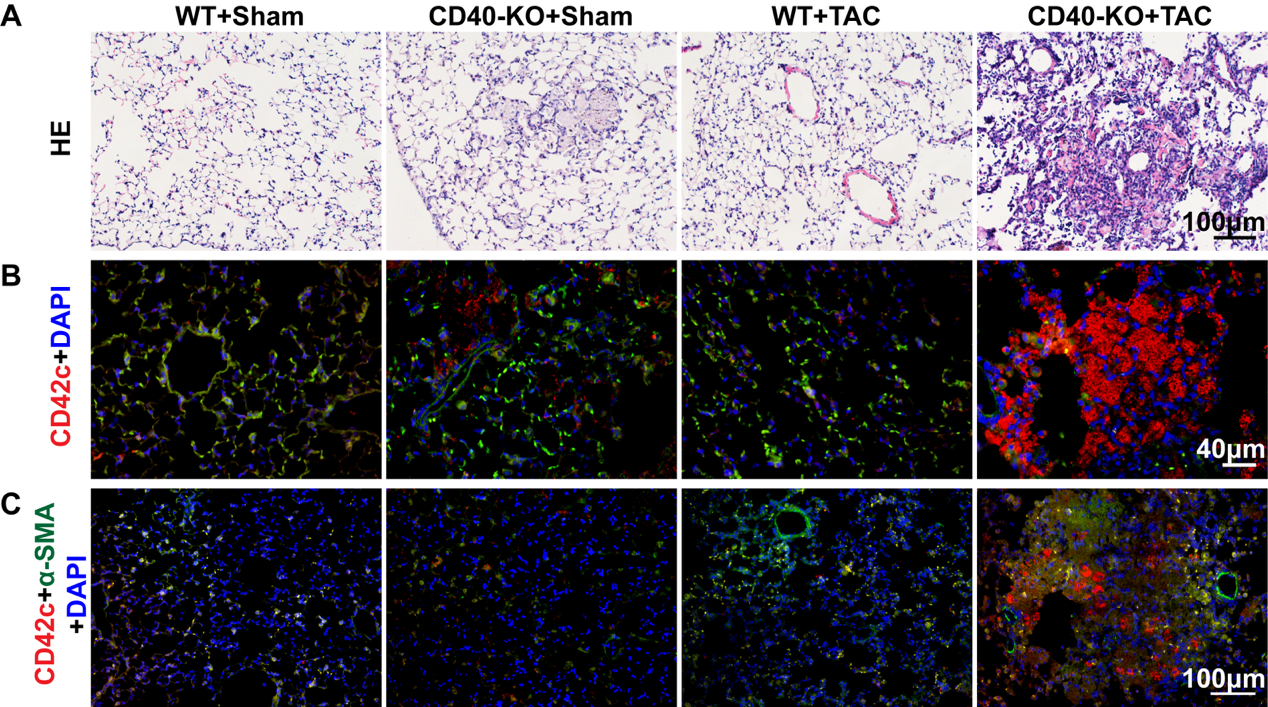


**Fig.S4 Representative HE and CD42c staining of pulmonary tissues in WT and CD40 KO mice showed drastic increase of CD42c+ staining in CD40 KO mice after TAC.** Representative images of HE staining in the lung (A). Immunofluorescence staining of CD42c (red) in lung tissues (B). Immunofluorescence co-staining of CD42c (red) and smooth muscle α-actin (green) in the lung (C).


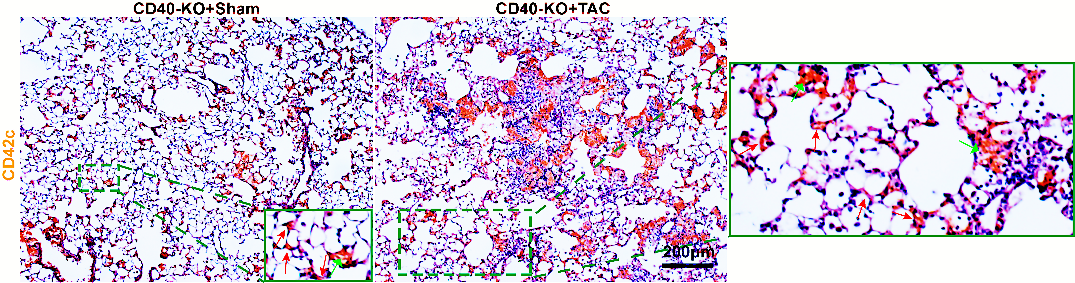


**Fig.S5 Histological straining of CD42c reveals pulmonary thrombosis in CD40 KO mice under sham or TAC conditions.** Immunohistochemical staining of CD42c in lung, red arrow indicated the thrombosis were mainly distributed inside micro vessel. Green arrow indicated the thrombosis distributed inside alveoli.

**
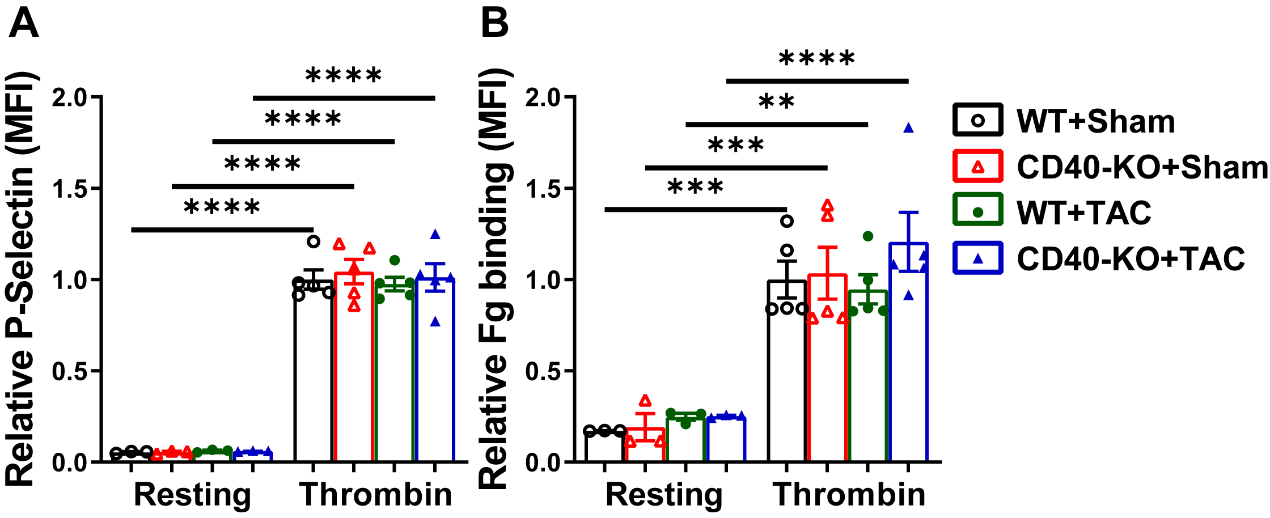
**

**Fig.S6 CD40 KO did not affect platelet p-selectin expression and fibrinogen binding at resting conditions and after thrombin stimulation.** MFI of P-selectin expression or Alexa 647-Fg binding on mouse platelets in response to 0.1 U/mL α-thrombin (n=3-5) (A, B). All quantitative data are reported as mean ± SEM. Data were analyzed using one-way ANOVA followed by Bonferroni post hoc analysis. **p*﹤0.05, ***p*﹤0.01, ****p*﹤0.001,*****p*﹤0.0001.

**
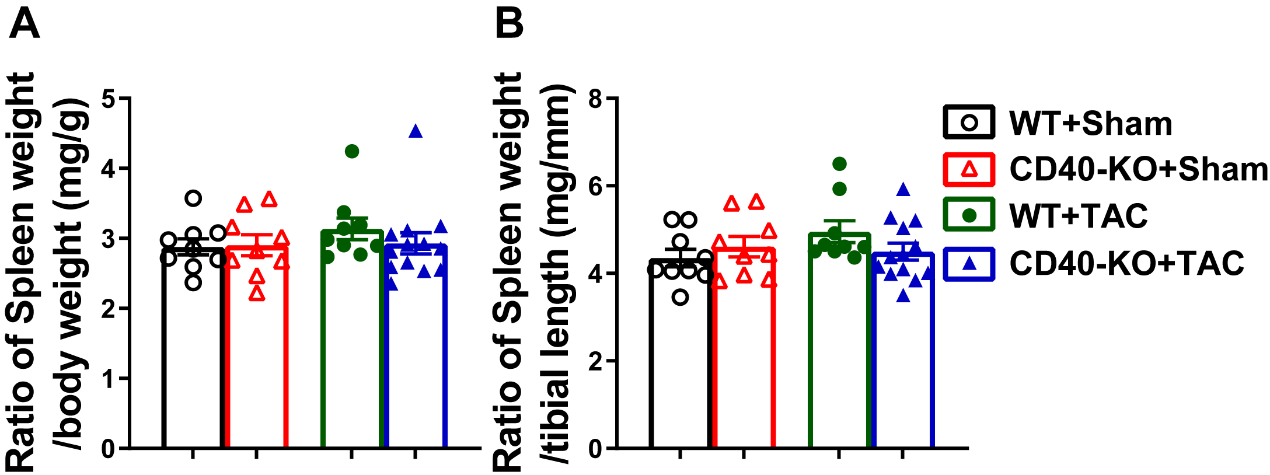
**

**Fig.S7 CD40 KO did not affect mouse spleen weight under control conditions or after TAC.** The ratio of spleen weight to bodyweight or tibial length of WT and CD40 KO mice under control or TAC condition (n=9-13) (A, B). All quantitative data are reported as mean ± SEM. Data were analyzed using one-way ANOVA followed by Bonferroni post hoc analysis.

**
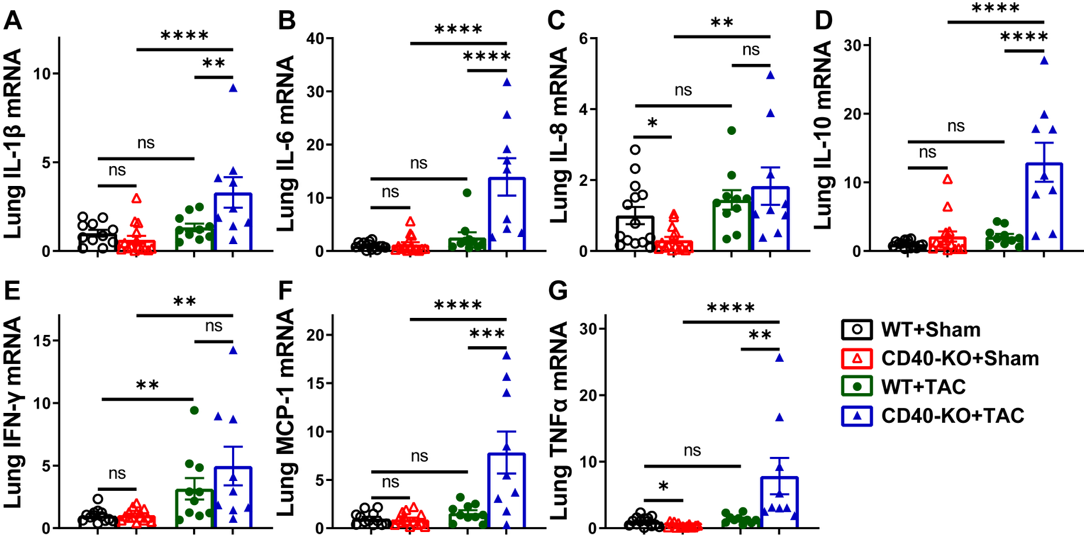
**

**Fig.S8 Real time PCR showed that TAC caused significant increases of pulmonary cytokines and adherence factor MCP1 in mice.** Quantitative RT-PCR results of IL-1β, IL-6, IL-8, IL-10, IFN-γ, MCP-1 and TNFα in lung lysates (n=9-15) (A-G). mRNA was normalized to 18S. All quantitative data are reported as mean ± SEM. Data were analyzed using one-way ANOVA followed by Bonferroni post hoc analysis. **p*﹤0.05, ***p*﹤0.01, ****p*﹤0.001,*****p*﹤0.0001.


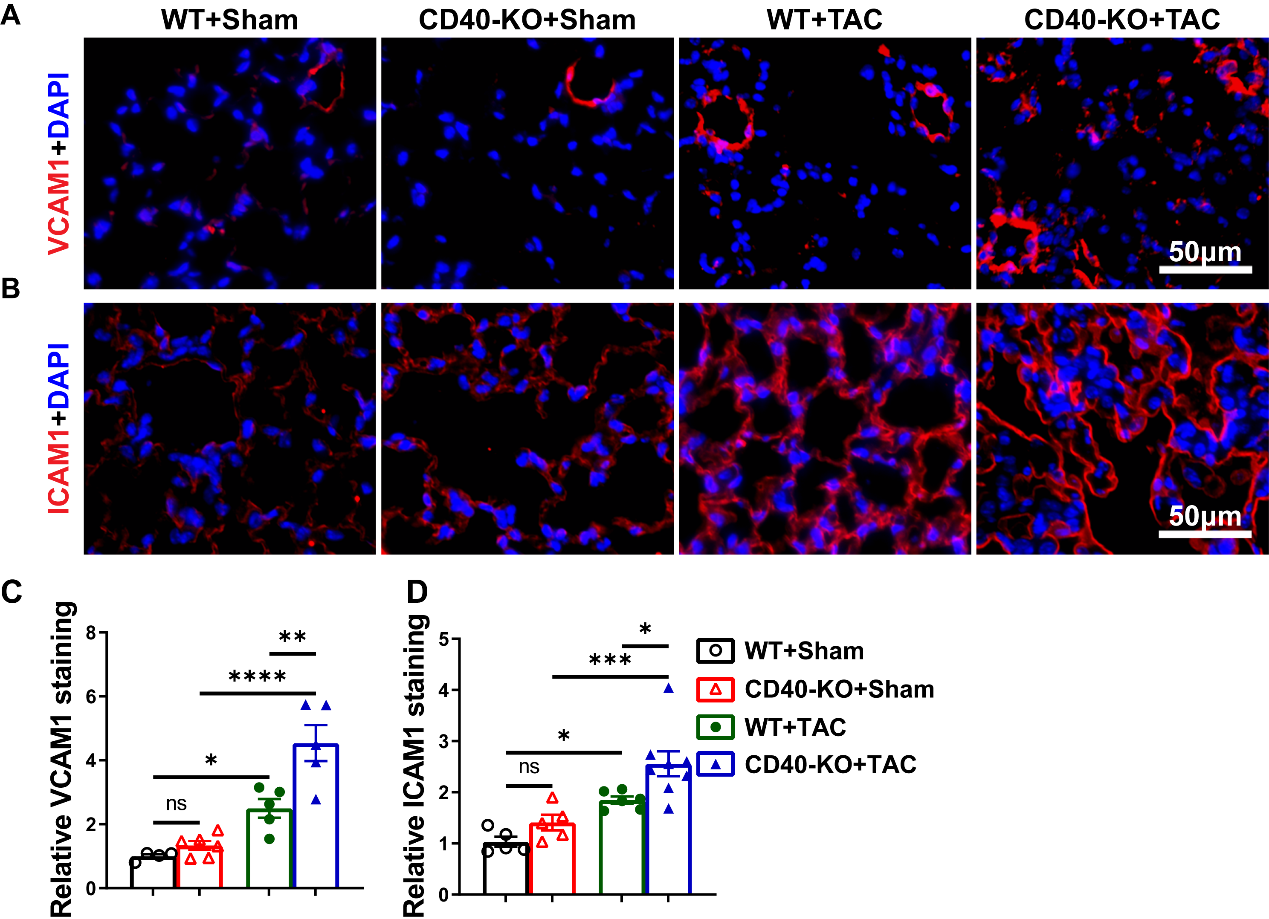


**Fig.S9 Immune histological staining demonstrated that CD40 KO significantly exacerbated TAC-induced pulmonary expression of VCAM1 and ICAM1.** Representative images of VCAM1 and ICAM1 staining in the lung (A, B). Quantitative data of VCAM1 and ICAM1 in the lung (C, D). n=4-8. All quantitative data are reported as mean ± SEM. Data were analyzed using one-way ANOVA followed by Bonferroni post hoc analysis. **p*﹤0.05, ***p*﹤0.01, ****p*﹤0.001,*****p*﹤0.0001.
